# Contaminated feed-borne *Bacillus cereus* aggravates respiratory distress post avian influenza virus H9N2 infection by inducing pneumonia

**DOI:** 10.1038/s41598-019-43660-2

**Published:** 2019-05-10

**Authors:** Qiang Zhang, Zonghui Zuo, Yongxia Guo, Tianyuan Zhang, Zhenhai Han, Shujian Huang, Musafiri Karama, Muhammad Kashif Saleemi, Ahrar Khan, Cheng He

**Affiliations:** 1 College of Life Science and Engineering, Foshan University, 528531 Foshan, China; 2 Key Lab of Animal Epidemiology and Zoonosis of Ministry of Agriculture, College of Veterinary Medicine, China Agricultural University, Beijing, 100193 China; 3 Faculty of Veterinary Science, University of Pretoria, Ondertepoort, 0110 South Africa; 4 Faculty of Veterinary Science, University of Agriculture,Faisalabad, 38040, Pakistan; 5 Shandong Vocational Animal Science and Veterinary College, Weifang, 261061 China

**Keywords:** Fungal biology, Infection, Bacterial infection

## Abstract

*Avian influenza virus* subtype H9N2 is identified in chickens with respiratory disease while *Bacillus cereus* (*B. cereus*) has been frequently isolated from chicken feed in China. However, the roles of co-infection with these two pathogens remain unclear. In the present study, SPF chicks were intragastrically administered with 10^8^ CFU/mL of *B. cereus* for 7 days and then inoculated intranasally with 100 EID_50_ of H9N2 three days later. Alternatively, chickens were initially inoculated with H9N2 and then with *B. cereus* for one week. Post administration, typical respiratory distress persisted for 5 days in both co-infection groups. Gizzard erosions developed in the groups *B. cereus*/H9N2 and *B. cereus* group on 7^th^ day while in group H9N2/*B. cereus* on 14^th^ day. More importantly, both air-sac lesions and lung damage increased significantly in the co-infection group. Significant inflammatory changes were observed in the *B. cereus* group from day 7 to day 21. Moreover, higher loads of H9N2 virus were found in the co-infected groups than in the H9N2 group. Newcastle Disease Virus (NDV) specific antibodies were decreased significantly in the H9N2/*B. cereus* group compared to the *B. cereus* and the *B. cereus*/H9N2 groups. Nonspecific IgA titers were reduced significantly in the *B. cereus* group and the H9N2/*B. cereus* group compared to the control group. In addition to this, lower lymphocyte proliferation was found in the con-infection groups and the H9N2 group. Hence, feed-borne *B. cereus* contamination potentially exacerbates gizzard ulceration and aggravates H9N2-induced respiratory distress by inhibiting antibody-mediated immunity and pathogen clearance. Thus controlling the *B. cereus* contamination in poultry feed is immediately needed.

## Introduction

*Bacillus cereus* (*B. cereus*) is among the microorganisms most often isolated from cases of food spoilage and gastrointestinal diseases as well as non-gastrointestinal infections due to its ability to produce several enterotoxins such as the heat-stable emetic toxin i.e., cereulide and tissue-destructive enzymes^1^. This opportunistic pathogen leads to vomiting and diarrhea syndromes in livestock and human beings, which is associated with rapidly fatal clinical infections, especially in neonates and immunocompromised individuals. Among cumulative food poisoning infections, as a cause it stands at third important position after *Salmonella* species and *Staphylococcus aureus*, and approximately from 10^2^ to 10^4^CFU/g has been reported in some food^2^. In a survey, contamination with 1.3 × 10^8^ CFU of *B. cereus* contributed to egg deterioration which was largely eliminated at low temperature^3^. A recent report indicated that *B. cereus* isolation rates in litter material, droppings, birds feed, liquid manure and raw milk was found to be 93.3%, 78.9%, 41.2%, 100.0% and 9.8%, respectively in local 10 dairy farms. Milk-borne *B. cereus* might represent a potential hazard to consumers due to inactivated during milk manufacturing^4^. On the contrary, improved immune status of piglets by *B. cereus var*. toyoi has been reported which could be by inducing a higher CD4+/CD8+ ratio and improving the functions of systemic immune cells^5^. *B. cereus* BC7 could efficiently detoxify zearalenone (ZEN) both *in vitro* and *in vivo*, indicating a potential feed additive for removing ZEN in a mouse model^6^.

Additionally, within the macrophages spores of *B. cereus* can survive, thus absconding this hostile environment. Escaping of *B. cereus* from macrophages could be by hijacking an active cellular process or causing the lysis of the cells, remains unknown, perhaps could be a cytotoxic factor in action^7^. Moreover, genetic background of *B. cereus* is nearly alike that of *B. anthracis* allowing it to escape the immune system^8^.

In current China poultry industry, co-infection of avian influenza H9N2 subtype virus with *Ornithobacterium rhinotracheale, Aspergillus fumigatu*s, and *Chlamydia psittaci* leads to unadorned pneumonia and increased mortality in SPF birds, threating to poultry health and leading to a huge economic loss^9^. A G57-genotype H9N2 emerged due to antigenic variation and facilitated adaptability in chickens. H9N2 was predominant in farms of vaccinated chickens in China, leading to outbreaks in 2010–2013^10^. In our pioneer study, both H9N2 virus and *B. cereus* have been frequently isolated from the lungs of birds showing respiratory distress while up to 80% of feed has been found contaminated with *B. cereus* (Unpublished). However, the pathogenic mechanism of any disease associated with *B. cereus* and H9N2 is unclear. In the present study, our hypothesis is that immune suppression by *B. cereus* causes aggravation of the respiratory distress after avian influenza subtype H9N2 infection. The experiments in our study were designed to evaluate both humoral and mucosal responses post inoculation with the combinations of *B. cereus* and H9N2 or H9N2 alone.

## Results

### The effect of *B. cereus*/H9N2 combined infection on gizzard erosion and weight gain

Identification of *B. cereus* C isolates was basis on biochemical tests and PCR assay. The DNA extracted produced the expected 1500 bp, 759 bp, 935 bp, 618 bp, 635 bp and 565 bp PCR products, respectively from 16SrDNA gene, *nheA* gene, *nheB* gene, *nheC* gene, *Em1*gene and *CytK* gene (Fig. S2). A sequence analysis of the 16S rDNA segment exhibited that this isolate sequence is 98–100% homologous with the reference strains of *B. cereus*^3^. The determined 16S rDNA sequence was submitted to GenBank (accession MK503979).

By day 3 post inoculation (PI), the infected chickens showed respiratory distress with open-mouth breathing and severe anorexia in groups H9N2/*B. cereus*, *B. cereus*/H9N2 and H9N2 alone. When compared to the breathing difficulty of birds in the H9N2 group on day 3, the breathing difficulty in birds inoculated with H9N2/*B. cereus* con-infection persisted for one week. By day 5, typical diarrhea was observed in groups *B. cereus* alone, *B. cereus*/H9N2 and H9N2/*B. cereus*. No birds died in any group during this study.

**Figure 1:**
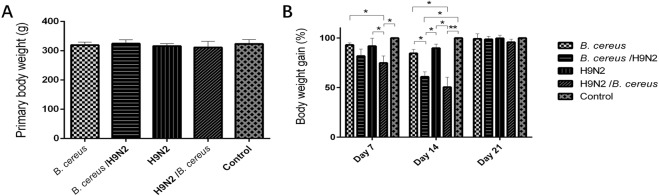
Weight gain post-inoculation with *B. cereus* or H9N2 virus. (**A**) No significant difference in initial weight gain was found among all the groups. (**B**) The mean weight gain was decreased significantly (*P* < *0.05*) in the H9N2/*B. cereus* group in comparison with the control group, the *B. cereus* group or the H9N2 group on day 7. On day 14 PI, the co-infection group weight gains were significantly less than the control group. On day 21 PI, non-significant differences were recorded among the groups.

Before treatment, the weight gains in all groups differed non-significantly (Fig. 1A). By day 7 PI, the weight gain was significantly (*P* < *0.05*) decreased in group H9N2/*B. cereus* in comparison with groups H9N2 or *B. cereus*. By day 14 PI, weight gain decreased significantly (*P* < *0.05*) in groups H9N2/*B. cereus* and *B. cereus*/H9N2 as compared to that of groups H9N2 or *B. cereus*. By day 21 PI, weight gain differed non-significantly among all groups (Fig. 1B).

**Figure 2:**
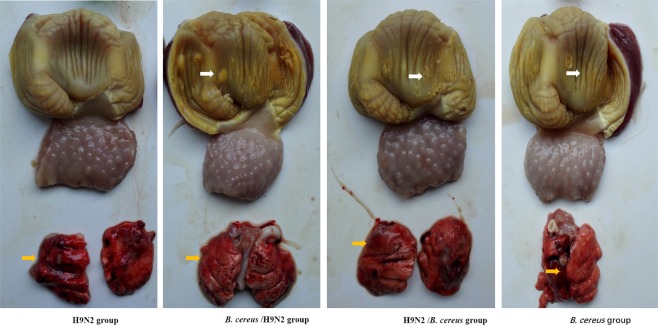
The effect of *B. cereus*/H9N2 co-infection on gizzard erosion and lung inflammation by day 7 PI. Birds inoculated with *B. cereus* or *B. cereus*/H9N2 displayed severe gizzard erosion and ulceration syndrome (GEU) while medium GEU was observed in the H9N2/*B. cereus* group (white arrows). Hemorrhagic lungs were observed in all the groups (orange arrows).

**Figure 3:**
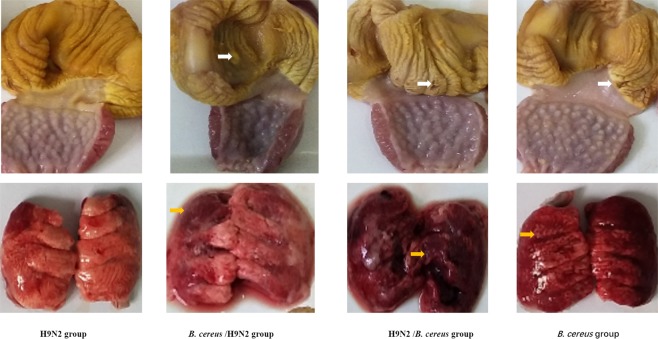
The effect of *B. cereus*/H9N2 co-infection on gizzard erosion and lung lesions by day 14 PI. Birds inoculated with *B. cereus*/H9N2 or H9N2/*B. cereus* displayed typical GEU and GEU recovery was observed in the *B. cereus* the group (white arrows). More interestingly, severe hemorrhagic lungs were observed in the H9N2/*B. cereus* group and fibrosis developed in the *B. cereus*/H9N2 group. Severity of lung lesions decreased in the H9N2 group (orange arrows).

At necropsy examination on day 7 PI, birds administered with *B. cereus* or *B. cereus*/H9N2 displayed the gizzard erosion and ulceration syndrome (GEU) on day 7 PI (Fig. 2). The severe lung hemorrhages were not only observed in groups H9N2 and H9N2/*B. cereus*, but also in the *B. cereus* group during all observations. On day 14, severe ulcerative lesions developed in groups H9N2/*B. cereus* and *B. cereus*. More importantly, hemorrhages were also widely evident in groups H9N2/*B. cereus* and *B. cereus* than those in the H9N2 group . The infected lungs were purple-red and condensed appearance than normal. The birds with *B. cereus*/H9N2 developed multiple foci of pulmonary consolidation in both lungs (Fig. 3).

### The effect of *B. cereus* and H9N2 con-infection on the respiratory tract

**Figure 4:**
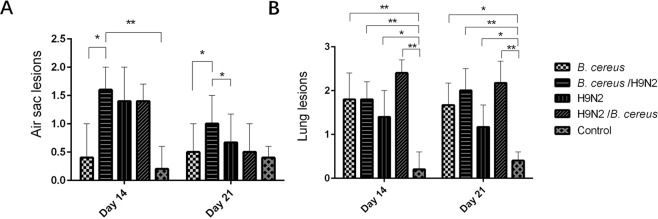
Effect of co-infection with *B. cereus* and AIV H9N2 on air sacs and lung lesions. (**A**) The air sac lesions were significantly increased in the *B. cereus*/H9N2 group compared to those in the control group or the *B. cereus* group on day 14 and day 21 (*P* < *0.05*) or H9N2 alone on day 21 (*P* < *0.05*). (**B**) Birds infected with *B. cereus* alone developed significant lung inflammation as compared to the groups H9N2 (*P* < *0.05)* and control (*P* < *0.01)*. Lung inflammation was significantly greater in the H9N2/*B. cereus* group compared to those of the *B. cereus*/H9N2 group, or *B. cereus* group *(P* < *0.05)* or H9N2 group or the control group *(P* < *0.01)* on day 21.

Birds in the *B. cereus*/H9N2, H9N2 and H9N2/*B. cereu*s groups had more severe air sac lesions when compared with those infected with *B. cereus* alone on day 14. On day 21, air sac lesions were more severe (*P* < *0.05*) in the *B. cereus*/H9N2  group as compared to the lesions present in other groups (Fig. 4A). In  the control group, no lung inflammation was noted while lung inflammation was significantly more severe in birds of all the infected groups. Lung inflammation was significantly (*P* < *0.01*) greater in the two co-infection groups as compared to the control group on days 14 and 21 (Fig. 4B).

**Figure 5:**
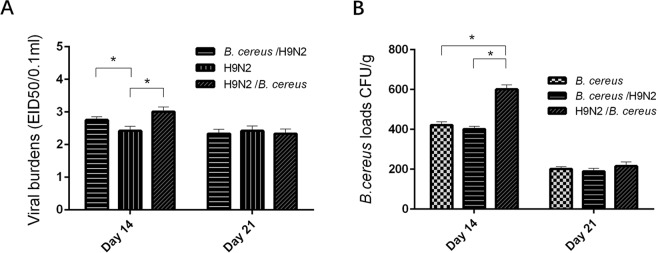
Lung pathogen loads. (**A**) Higher mean H9N2 virus loads were found in the *B. cereus*/H9N2 and H9N2/*B. cereus* groups compared to those of the H9N2 group on day 14 PI (*P* < 0.05). (**B**) The mean *B. cereus* loads were significantly greater in the *B. cereus* and *B. cereus*/H9N2 groups than in the H9N2/*B. cereus* group on day 7 PI (*P* < *0.01)* but higher in the H9N2/*B. cereus* group on day 14 (*P* < *0.05)*. No significant differences were found on day 21 between groups in H9N2 loads or *B. cereus* clearance.

The H9N2 group exhibited significantly lower (*P* < *0.05*) burden of the H9N2 virus than in either of the two co-infected groups on day 14. However, a lower (*P* < *0.05*) H9N2 virus burden was still evident in the *B. cereus*/H9N2 group than that of the H9N2/*B. cereus* group. While non-statistical difference in viral burden among the three groups on day 21 was found (Fig. 5A). A significant (*P* < *0.05*) increase of the *B. cereus* load was recorded in the H9N2/*B. cereus* group compared to other two groups on day 14 while on day 21, the *B. cereus* load was non-significant difference among the three groups (Fig. 5B).

### The effect of *B. cereus* and H9N2 co-infection on NDV-specific antibody and IgA response

**Figure 6:**
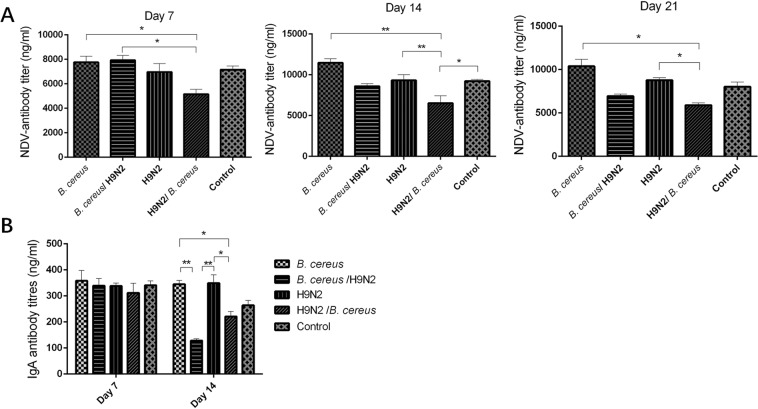
Effect of *B. cereus* and H9N2 co-infection on NDV-specific antibodies and IgA antibodies. (**A**) NDV-specific antibodies decreased significantly in the H9N2/*B. cereus* group compared to the other infected groups on day 7 (*P* < 0.05). On day 14 the NDV antibody response in the H9N2/*B. cereus* group was lower than in the control, *B. cereus* or *B. cereus*/H9N2 groups (*P* < 0.05). By day 21 PI, NDV-specific antibodies decreased significantly in the H9N2/*B. cereus* group when compared to the control, *B. cereus* or *B. cereus*/H9N2 groups (*P* < *0.05*). (**B**) Non-specific IgA antibody levels were decreased significantly in the H9N2/*B. cereus* group in comparison with other groups from day 14 to day 21 (*P* < *0.05*).

Following vaccination, NDV-specific antibodies increased significantly in the groups H9N2/*B. cereus* (*P* < *0.05*) and *B. cereus* (*P* < *0.01*) in comparison with  those of the *B. cereus*/H9N2 group at three-time points. A statistically significant decline in NDV-specific antibody levels was consistently noted in the H9N2/*B. cereus* group as compared to the *B. cereus* group from day 7 to day 21 (*P* < *0.05*) (Fig. 6A). IgA antibody levels were significantly lower in the co-infected animals than in bird infected with only one agent (*P* < *0.01*) (Fig. 6B).

### The effect of concurrent infection with *B. cereus* and H9N2 on lymphocyte proliferation

**Figure 7:**
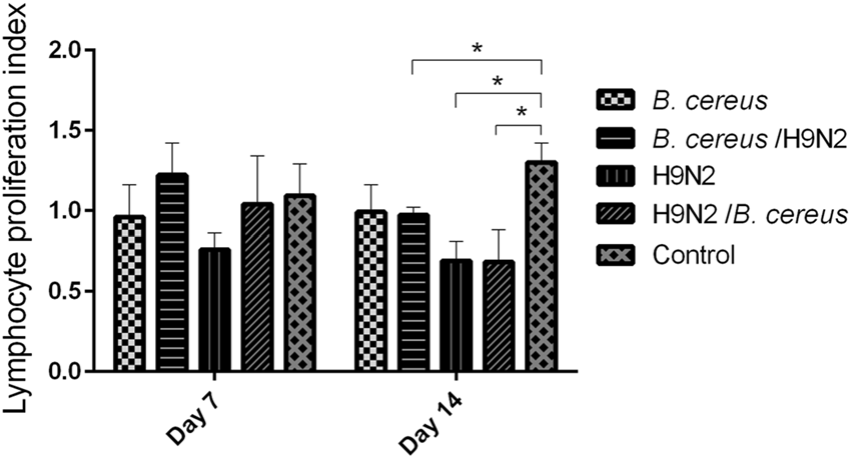
Effect of *B. cereus* and H9N2 co-infection on lymphocyte proliferation. By day14 PI, lymphocyte proliferation index was significantly reduced in the H9N2 group or H9N2/*B. cereus* group compared to the control group or the *B. cereus* group (*P* < *0.05*). As compared to the control group, birds with *B*. *cereus* inoculation produced a lower level of lymphocyte proliferation index. No statistical difference was found between the *B. cereus* group and the *B. cereus*/H9N2 group.

On day 7, the H9N2 group exhibited lower proliferation values when compared to the other groups, including the control group, although these differences were not statistically significant. On day 14 PI, birds in H9N2/*B. cereus* and H9N2 group had a significantly (*P* < *0.05*) decreased lymphocyte proliferation index than in the birds of control or *B. cereus*/H9N2 group (Fig. 7).

### The effect of concurrent with *B. cereus* and H9N2 on cytokine secretions

**Figure 8:**
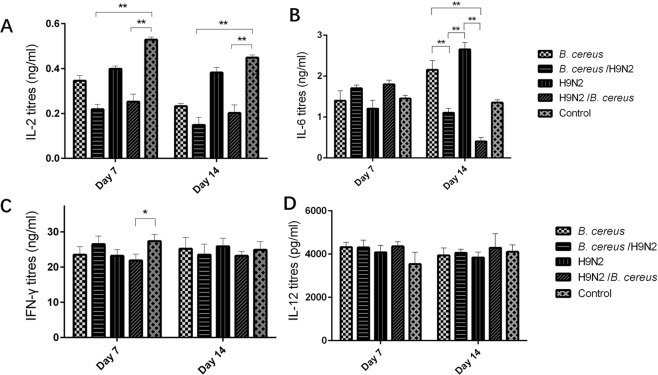
Effect of *B. cereus* and H9N2 co-infection on cytokine secretions. (**A**) IL-2 expression was reduced significantly in the *B. cereus*/H9N2 group and H9N2/*B. cereus* group compared to that of the control group on day 7 and day 14 *(P* < *0.01)*. (**B**) A significant decrease of IL-6 was detected both in the *B. cereus*/H9N2 group and the H9N2/*B. cereus* group compared to other groups on day 14 (*P* < *0.05*). The greatest reduction in IL-6 occurred in the H9N2 group on day 14. (**C**) IFN-γ expression was decreased in the H9N2/*B. cereus* group in comparison to the control group on day 7 PI *(P* < *0.05)* and no significant difference was found between groups on day 14 PI. (**D**) No significant difference in IL-12 was detected between groups on day 7 and day 14.

Regarding cytokine expression, on days 7 and 14 both the *B. cereus*/H9N2 group and H9N2/*B. cereus* group exhibited a significant (*P* < *0.01*) decreased in IL-2 expression in comparison to that of the controlgroup or H9N2 group. Moreover, IL-2 expression was reduced significantly (*P* < *0.05*) in the *B. cereus* group as compared to that of the control group. Whereas, non-significant difference between two co-infected groups was found (Fig. 8A). In addition to IL-2 levels, IL-6 reduced significantly (*P* < *0.05*) in both the *B. cereus*/H9N2 and the H9N2/*B. cereus* groups compared to the *B. cereus* or H9N2 group on day 14 (Fig. 8B). On day 7, the IFN-γ expression was significantly higher in the control group or *B. cereus*/H9N2 group as compared to the H9N2/*B. cereus* group (Fig. 8C). No statistical differences in IL-12 were found between groups on days 7 and 14 (Fig. 8D).

## Discussion

In the present study, birds orally administered *B. cereus* exhibited the gizzard erosion and ulceration syndrome (GEU), and haemorrhagic inflammation in the lungs. A primary infection with H9N2 followed by inoculation with *B. cereus* caused birds to develop more severe breathing difficulty in comparison with H9N2 infection alone. More interestingly, higher lesion scores in the air-sacs and lungs were diagnosed in groups *B. cereus*/H9N2 and H9N2/*B. cereus* while H9N2 virus concentrations were significantly higher in the groups *B. cereus*/H9N2 and H9N2/*B. cereus* than in the H9N2 group on day 14. The above facts warrant our hypothesis that primary contagion with *avian influenza virus* subtype H9N2 followed by *B. cereus* inoculation aggravates avian respiratory distress.

During seasonal outbreaks of avian influenza, secondary bacterial infections are favored by the changes in respiratory tract epithelium^11,12^, resident macrophages^13^, and recruited white blood cells due to the direct virus cytopathic effects^14^. The performed experimentations in our study were planned to identify the pathogenesis of *B. cereus* under such conditions. In current study, chickens received orally 10 × 8 CFU/ml of *B. cereus* for 7 consecutive days, which was comparable to the administration of the fast-growing broiler per day. The high inoculation is based on 100 g feed consumption per SPF chicken on day 21. In our preliminary survey, average colonies of *B. cereus* were determined to be 1 × 10^6^ CFU per gram in the feed. Main cause of complications and mortality following an *avian influenza virus* attack, are the secondary bacterial infections. Combined infection with *influenza A virus* from human infection (4% of the viral lethal dose 50%) and *Bacillus thuringiensis* (10^7^ spores) from a commercial source leading to 100% mortality in mice indicates that non-pathogenic *Bacillus* species will pose a high risk during bird flu outbreak^15^.

In the current study, no chickens died when inoculated with the co-infection of AIV H9N2 with *B. cereus* or AIV H9N2 alone, but a severe fibrinous exudate, typical pneumonia, GEU were detected in the groups *B. cereus*/H9N2, H9N2/*B. cereus* and *B. cereus* alone. This suggests that *B. cereus* administration produced significant gizzard erosions and lung injury, while co-existence with H9N2 aggravated such respiratory disease. The present data showed that the most severe lung lesions were observed in the groups *B. cereus*/H9N2, H9N2/*B. cereus* and *B. cereus*, indicating that hemorrhagic inflammations is induced by *B. cereus* contamination in the poultry industry.

As *Bacillus cereus* infection results in gizzard erosions and lung lesions, it might contribute to damage to the digestive tract and respiratory epithelial cells, facilitating infection by other pathogens. The mucosal immune system must balance tolerogenic responses against respiratory tract commensal microbiota, while preserving the ability to support protective inflammatory responses against attacking pathogens. This delicate balance is maintained through the harmonized signals and interactions of a variety of specialized cells^16^. Besides providing an important barrier between invading pathogens and the underlying tissue, lung epithelial cells can interact with bacteria via both macrophages and antibacterial peptides. Once the respiratory mucosal barrier is damaged, infection by other pathogens will be facilitated^17^. In the present study, non-specific IgA levels were decreased significantly in the *B. cereus* group and H9N2/*B. cereus* group at two-time points, suggesting mucosal immune suppression. IgA protects the mucosal surface against bacterial destruction and lower IgA levels would be expected to facilitate development of gizzard ulceration and hemorrhagic pneumonia in the *B. cereus* group and H9N2/*B. cereus* group. Also, low levels of IgA might favour *B.cereus* and H9N2 survival in the lungs of chickens.

Secondly, the dysfunction of antibody-mediated immunity is associated with poor development of NDV-specific antibodies, characterized as the lower IgG response that was found in the H9N2/*B. cereus* group on days 7, 14 and 21. Consequently, low IgG-specific antibody leads to vaccine failure and NDV outbreak in poultry flock. This might be associated with numerous NDV cases in broilers post immunization with live attenuated vaccine. Both air-sac lesions and lung damage were found significantly higher in the birds in the groups *B. cereus/*H9N2 and H9N2*/B. cereus* as compared to the control group. Aforementioned findings suggest that *B. cereus* might facilitate H9N2 infection, leading to vaccine failure. The present results indicate that both *B. cereus/*H9N2 and H9N2*/B. cereus* inoculation protocols induced lower lymphocyte proliferations on day 14. The fact that birds in the co-infection groups exhibited severe air-sac lesions and lung injury suggests a poor innate immune response against infection of H9N2 in the lungs. In general, host defence and Th1 responses are more effective against intracellular pathogens (viruses and bacteria that are inside host cells)^18^. Regarding cytokine secretions, co-infection of *B. cereus* and H9N2 induced lower IL-2 and IFN-γ while IL-6 levels were upregulated in contrary to those of the control group, indicating imbalance in the immune response of Th1/Th2 and the reduced IL-2 in the groups initially infected with *B. cereus* might facilitate AIV H9N2 to survive and more severe lesions in the lungs due to Th1/Th2 imbalance. The high concentration of IL-2 is associated with CD8+ T cells expansion and differentiation in conforming to the antiviral immune response, and the reduced IL-2 concentration might lower the capacity to control the virus load^19^. This might account for the obstinate avian airsacculitis and failure of vaccination plans in the poultry industry. However, no significant difference of IL-12 was found among all the groups from day 7 to day 14. Post administration with IL-12 in mammals, NK cells were stimulated to produce IFN-γ through their constitutive mien of IL-12R^20^ and production of IFN-γ by IL-12 was in a dose-dependent manner^21^. In this study, no significant difference of IFN-γ was found among the *B. cereus* group, H9N2 group or combination of two pathogens due to IL-12 secretion.

Importantly, food-borne *B. cereus* might pose a risk for human health. H9N2 influenza virus is not limited to birds and frequent cases of humans suffering with H9N2 virus infections are on the recorded^18,22^. H9N2 influenza virus is distinct from H5 and H7 influenza viruses, some of these are exceptionally pathogenic, could be due to the multiple basic amino acids presence in the hemagglutinin (HA) cleavage site as defined by the Office International des Épizooties (OIE). All of the isolated H9N2 strains have low pathogenicity in accordance with the OIE cataloging. In spite of this fact, H9N2 virus infections have caused serious disease in several cases, even high mortality in some cases in domestic poultry have been reported^11,13,23^. On the other hand, consumers have easy access to food-borne *B. cereus* pathogens via contamination, particularly the biotype of *B. cereus* infection, which would expect to aggravate H9N2 respiratory diseases by suppressing the host immune response, potentially leading to a human pandemic during a seasonal influenza outbreak. This is the first study to reveal that *B. cereus* aggravates H9N2 infection in chickens. Further studies should check whether *B. cereus-*mediated lung inflammation contributes to respiratory diseases with other pathogens, generation of H9N2 mutants and vaccination failure in the poultry industry.

In conclusion, *B. cereus*, as a primary or an enduring latent infection can cause lung inflammation *in vivo*, and may increase other-pathogens susceptibility, such as H9N2. Our findings suggest that H9N2/*B. cereus* infection contributes to NDV vaccine failure and severe respiratory distress by eliciting a damaged cellular immunity.

## Materials and Methods

### Animals

Five 10-day-old SPF embryonated eggs and 105 SPF birds of 21 days age were purchased from Weitong Merial Laboratory Animal Co., Ltd, Beijing and kept at the Experimental Center for Animals, China Agricultural University (CAU), Beijing, China. All birds were kept in strict accordance with the Regulations for the Administration of Affairs Concerning Experimental Animals of the State Council of the People’s Republic of China. The experimental protocols were approved by the Committee for Experimental Animal Management of CAU and followed humane protocols to minimize animal pain as described previously^24,25^. Briefly, the birds were monitored 2 or 3 times daily for clinical symptoms and chickens displaying clinical signs were euthanized. Birds were euthanized with an overdose of CO_2_ using a gradual fill (30% chamber volume/min) device. The CO_2_ movement was continued for a minimum of 1 min after the loss of respiratory signs. When death was confirmed, an additional secondary physical euthanasia (i.e. cervical dislocation) was carried out before sampling and carcass discarding.

### Pathogen isolates

Lungs were collected aseptically from the diseased chickens. Streak cultures were performed using Mannitol Yolk Polymyxin (MYP) (Beijing Land Bridge Technology Co., LTD, China) and incubated at 37 °C under aerobic conditions for 24 h. The colony of interest was identified by Gram stain and biochemical assays. Biochemical characteristics of the bacterial culture, as well as molecular identification of *B. cereus* C type were then carried out employing 16SrRNA gene, *nheA* gene, *nheB* gene, *nheC* gene, *Em1*gene and *CytK* gene. Additionally, the 16SrRNA gene sequence was analyzed by a commercial institute (Beijing Genomics Institute, China). H9N2 AIV/chicken/Shandong/2011 was isolated from broilers as described formerly^21^. Due to the potential for aerosol infection of avian influenza A H9N2 for human, respiratory protection was required for all activities conducted at Biosafety Level 2 (BSL-2).

### Animal studies

One hundred and five SPF chickens were randomly divided into 5 groups with 21 chickens per group (Supplemental Fig. 1). Before the experiment, all the birds received intranasally the attenuated vaccine against NDV (M/S Ceva-Huadou Co. Ltd, Beijing, China), one dose per chicken, followed by post immunization with attenuated NDV vaccine 3 days later, *B. cereus* group birds were inoculated intragastrically with 1 mL (1 × 10^8^ CFUs/mL) of the liquid culture of *B. cereus* each day for 7 consecutive days. H9N2 group birds were inoculated intranasally (it) with 0.2 mL 100 EID_50_ H9N2 virus. *B. cereus*/H9N2 group birds received intragastrically with 1 × 10^8^ CFUs of *B. cereus* for 7 days and then 100 EID_50_ H9N2 virus in 0.2 ml via the intranasal route H9N2/*B. cereus* group birds were administered intranasally with 100 EID_50_ of H9N2 virus, and then administered 1 × 10^8^ CFUs/ml of *B. cereus* intragastrically for one week. Control group birds were intranasally treated with the same amount of sterile physiological saline. All animals were given SPF feed free of *B. cereus*. Clinical signs/symptoms, body weight, and mortality were recorded a minimum of twice daily and the monitoring period continued till the end of the experiment.

### Determination of NDV-specific antibodies, IgA, lymphocyte proliferation and cytokine levels

Seven blood samples per group were collected from jugular vein punctures at days 7, 14 and 21 post-infection (PI), and NDV-specific antibodies were measured using a commercial kit (IDEXX, USA). In addition to weight gains, 8 peripheral blood lymphocyte proliferations were determined on day 7 and day 14 using a BrdU cell proliferation ELISA kit (Abcam, Beijing, China)^25^. Six bronchial alveolar lavage fluid (BALF) samples were examined as previously reported^26^. Briefly, 6 chickens were euthanized at days 7 and 21 post-infection and the lungs were lavaged three times with 1.0 mL sterile saline. Suspensions were centrifuged at 1000 rpm for 5 min and stored at −80 °C until used. The concentration of IgA of the BALF was measured using an ELISA kit (Abcam, Beijing, China). The concentrations of IL-2, IL-6, IL-12 and IFN-γ from 6 BALF samples per group were determined using commercial kits (Kingfisher Inc, USA).

### Air-sac lesions and lung index

Seven birds from each group were euthanized post-anesthesia by CO2 inhalation on day 14 and day 21 PI. The air-sac and lung lesions were determined as previously described^26,27^ (Tables 1 and 2).

**Table 1:** Scoring of air sac lesions in chickens.

Assigned score	Air sac lesions
0	Normal, clean, thin and transparent
1	Slightly thickened and slightly turbid, or individual local white exudate
2	Grayish white exudate at a few places of the air sac, moderate sac thickening
3	Majority of the air sacs were fully covered with yellow white caseous exudate and obvious thickening air sacs
4	Serious air sac lesions with white thick exudate on thoracic cavity and abdominal cavity

**Table 2:** Scoring of lung lesions in chickens.

Assigned score	Lung lesions
0	None
1	Slight edema of the alveolar walls
2	Moderate edematous thickening of alveolar walls with occasional alveoli contained coagulated edema fluid
3	Extensive occurrence of alveolar and interstitial edema

### Lung pathogen loads

The lungs were aseptically removed on days 14 and 21, respectively. Briefly, sterile lung tissues of 7 birds from each group were taken and then minced, 50 mg lung tissues from each bird and supernatants from lung homogenates were obtained. The homogenates were stored at 4 °C for 40 min and then centrifuged at 2000 rpm/min for 5 min. The supernatants were divided into two aliquots and maintained at −80 °C until used. One stock was used to determine the concentration of H9N2 virus by inoculating into five 10-day-old SPF embryonated eggs and the eggs were examined for 48 h. Thereafter, HI titer of allantoic fluid from each egg was tested to determine the 50% egg infective dose (EID_50_). For *B. cereus* determination, the samples from the lungs were inoculated onto an MYP agar plate (containing 50 μg/g of Colistin sulfate B) and incubated for 24 h at 37 °C. Finally, the number of bacterial colonies was measured.

### Statistical analysis

Data are expressed as mean ± SEM (standard error of the mean) and analyzed using STATISTCA v.7 (Stat Soft) software. Nonparametric analysis and Mann–Whitney U tests were performed for comparison between groups and the data presented as median values. Multiple group analysis, including the multiple comparison correction (Bonferroni) was carried out. Statistically significant differences were judged as *P* < *0.05*.

### Ethics approval

All experimental protocols were approved by the Committee on the Ethics of Animal Experiments of China Agricultural University (Permit Number: 20151110–160). This study does not involve the use of human sample or tissue.

## Supplementary information

Supplementary information

## Data Availability

The datasets used and/or analyzed during the current study are available from the corresponding author on reasonable request.
