# Supplementary material for: Contaminated feed-borne Bacillus cereus aggravates respiratory distress post avian influenza virus H9N2 infection by inducing pneumonia
Source: Sci Rep. 2019 May 10;9:7231. doi: 10.1038/s41598-019-43660-2 (PMC6510747; doi:10.1038/s41598-019-43660-2)
Supplement: Supplementary file 1 — Supplementary information [file 41598_2019_43660_MOESM1_ESM.pdf]

Contaminated feed-borne *Bacillus cereus* aggravates respiratory distress post avian influenza virus H9N2 infection by inducing pneumonia

Qiang Zhang[1,2], Zonghui Zuo[2], Yongxia Guo[2], Tianyuan Zhang[2], Zhenhai Han[2], Shujian Huang[1], Musafiri Karama[3], M Kashif Saleemi[4], Ahrar Khan[4]*, Cheng He[1,2]*

1. *College of Life Science and Engineering, Foshan University, Foshan 528531, Guangdong, China*
2. *Key Lab of Animal Epidemiology and Zoonosis of Ministry of Agriculture, College of Veterinary Medicine, China Agricultural University, Beijing 100193, China*
3. *Faculty of Veterinary Science, University of Pretoria, Ondertepoort 0110, South Africa*
4. *Faculty of Veterinary Science, University of Agriculture, Faisalabad, Pakistan*

Supplemental Figures

Fig. S1

One hundred and five 21-day-old SPF chickens were randomly divided in 5 groups with 20 chickens each group. Birds were inoculated intragastrically (it.) with 1 ml (1×10^8 CFUs/mL) of the liquid culture of *B. cereus* each day for 7 consecutive days, therefore. *cereus* group. Birds were inoculated intranasally (it) with 0.2 ml 100 EID_{50} H9N2 virus, therefore as H9N2 group. Chickens received 1×10^8 CFUs it. *B. cereus* for 7 days and then 100 EID_{50} H9N2 virus in 0.2 ml via intranasal way, *B. cereus* /H9N2 group. Birds were inoculated intranasally with 100 EID_{50} of H9N2 virus, and then administered 1×10^8 CFUs/mL of *B. cereus* via oral administration for one week, H9N2/*B. cereus* group. Body weight gains were monitor weekly and 10 blood samples were collected each week. NDV-specific antibodies, IgA, cytokines and splenic lymphocyte proliferations were determined using commercial kits. Six birds from each group were euthanized post-anesthesia by ether on day 14 and day 21 PI. The lesions of air-sacs and lungs were determined as previously described [22, 24] (Table 1 and Table 2).

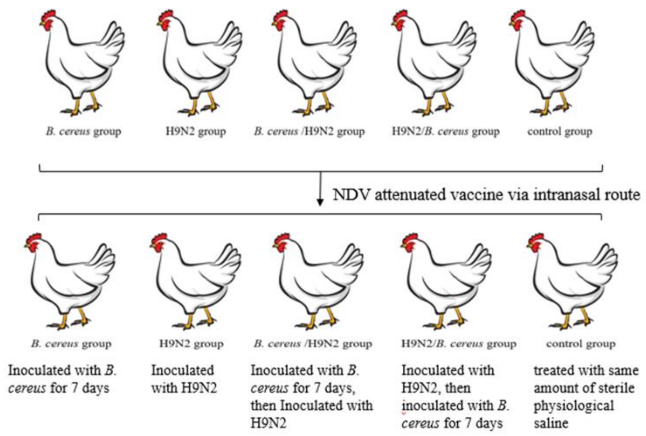

	Body weight	Gizzard erosion	Air sac lesions and lung inflammations	Pathogen loads in lungs	NDV-specific antibodies titre	Non-IgA titre	lymphocyte proliferation	cytokines (IL-2, IFN-γ ,IL-12 and IL-6)
Day 7	✔	✔			✔	✔	✔	✔
Day 14	✔	✔	✔	✔	✔	✔	✔	✔
Day 21	✔		✔	✔	✔			

Fig. S2

16s rRNA gene, *nheA* gene, *nheB* gene, *nheC* gene,*Em1gene* and *CytK* gene of *B. cereus* C type were confirmed to be 1500bp, 759bp, 935bp, 618bp, 635bp and 565bp, respectively using PCR method.

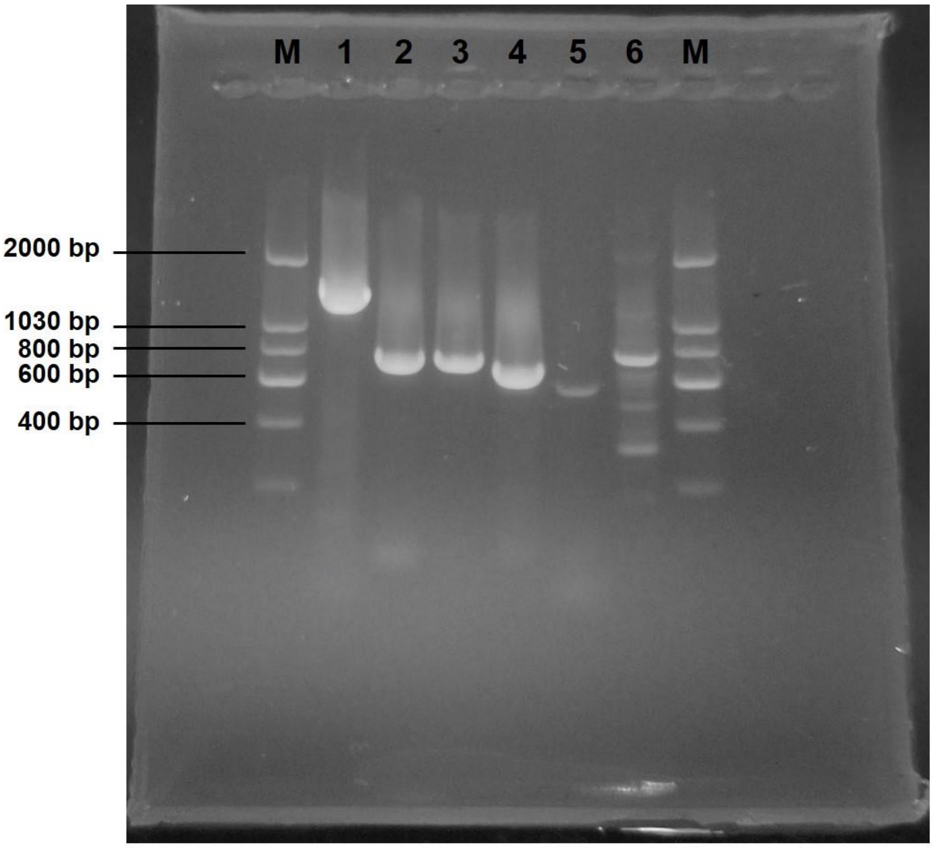

M: Marker; 1: 16s rDNA; 2: *nheA*; 3: *nheB*; 4: *nheC*; 5: *Em1*; 6: *CytK*
